# Ameliorative effects of camel milk and silymarin upon aflatoxin B1 induced hepatic injury in rats

**DOI:** 10.1038/s41598-023-41586-4

**Published:** 2023-09-12

**Authors:** Nahla H. Hassaneen, Shabaan A. Hemeda, Abeer F. El Nahas, Sabreen E. Fadl, Eman M. El-diasty

**Affiliations:** 1 Department of Animal Husbandry and Animal Wealth Development, Faculty of Veterinary Medicine, Matrouh University, Matrouh, Egypt; 2 Department of Animal Husbandry and Animal Wealth Development, Faculty of Veterinary Medicine, Alexandria University, Alexandria, Egypt; 3 Biochemistry Department, Faculty of Veterinary Medicine, Matrouh University, Matrouh, Egypt; 4 Mycology and Mycotoxins Department, Animal Health Research Institute (ARC), Giza, Egypt

**Keywords:** Biochemistry, Biotechnology

## Abstract

Aflatoxin B1 (AFB1) poses a major risk to both human and animal health because it contaminates food, feed, and grains. These dangerous effects can be mitigated using natural components. The purpose of this study was to examine the ameliorative effects of camel milk and silymarin supplementation upon aflatoxin B1 induced hepatic injury in rats. This improvement was assessed by measuring leukocytic and deferential counts, serum biochemical parameters, and gene expression of Tumor Necrosis Factor (*TNF-α*), antioxidant gene (NAD(P)H quinone oxidoreductase 1 (*NQO1*)), and base excision repair genes (*APE1* and *OGG1*) in the liver tissue, in addition to liver histopathology. Sixty mature males Wister white rats were used to perform the present study; the rats were distributed in six groups (ten rats/group). The control group (without any treatment) received saline by gavage. The camel milk group received 1 ml of camel milk/kg body weight. The silymarin group received 1 ml of silymarin suspension solution at a dose of 20 mg of silymarin/kg of b.wt. The aflatoxin group received an aflatoxin-contaminated diet at a dose of 1.4 mg of aflatoxin /kg of diet and received saline. The camel milk + aflatoxin group received the same previous oral doses of camel milk and an aflatoxin-contaminated diet at the same time. The silymarin + aflatoxin group received the same previous doses of silymarin orally and an aflatoxin-contaminated diet at the same time. The obtained data indicated the deleterious effect of aflatoxin B1 on the leukocytic count, activity of AST and ALT, serum proteins, ferritin, alpha-fetoprotein, carcinoembryonic antigen, liver pathology, and the expression of the studied genes. However, these deleterious effects were mitigated by camel milk and silymarin supplementation. Thus, we could conclude that the ingestion of camel milk and silymarin mitigated the negative effects of AFB1 on the hematology, activity of AST and ALT, serum proteins, ferritin, alpha-fetoprotein, carcinoembryonic antigen, liver pathology, and gene expression in the rat model.

## Introduction

Mycotoxins are byproducts of fungal metabolism that have adverse effects on human and animal health. These substances are frequently present in food, particularly in cases when harvest storage or transportation procedures are subpar^1^. According to estimates, mycotoxins are present in around 25% of all foods consumed worldwide^2^. Aflatoxins stand out among mycotoxins because of their ability to cause cancer. Several foods, including corn, peanuts, and others, contain aflatoxins (AFs), which are mostly generated by the fungus *Aspergillus spp.*^3^. Even though AFs aren't present in every fungus-contaminated food, they do exist everywhere since temperature, and humidity are ideal conditions for *Aspergillus* species to grow and produce these mycotoxins^4^. The most of *Aspergillus* species are soil fungi or saprophytes, although a small number can also cause plant illness, invasive disease in humans and animals, and storage rot. Corn, peanuts, cottonseed, rice, tree nuts, cereal grains, and fruits are some of the major agricultural commodities that are impacted by fungus growth and mycotoxins before or after harvest^5^. Aflatoxin B1 (AFB1), which has the highest potential for cancer development, is one of the four primary forms of aflatoxins present in foods, along with variations B2, G1, and G2. Aflatoxin M1 (AFM1), a byproduct of aflatoxin B1 metabolism in the animal body, is notable for both its ability to cause cancer and for being excreted in both animal and human milk^6^. It is well recognized that AFB1 has the potential to be genotoxic, mutagenic, immunogenic, and hepatotoxic, and that it may quickly harm the liver when consumed in significant doses^7^.

The significant concentration of antioxidants in natural foods and plants (carotenoidic, phenolic, flavonoid, anthocyanin derivatives, unsaturated fatty acids, vitamins, enzymes, and cofactors) has sparked interest in employing these substances in preventative and therapeutic phytotherapy in recent years^8^. In recent years, interest in camel milk and milk products has increased around the world due to the superior nutritional value and purported medicinal properties of the milk against a variety of human ailments^9^. Camel milk has greater nutritional value and is said to provide therapeutic benefits for some human diseases, including those related to diabetes, autism, antimicrobials, hypertension, cancer, cholesterol, and hypertension, as well as effects on hepatoprotection, hypoallergenicity, and immunological boosting^10^. Beta-caseins, milk whey proteins, including lactoferrin, lysozyme, lacto peroxidase, alpha-lactalbumin, and immunoglobulin, as well as vitamin C and lactic acid bacteria (LAB), are all abundant in camel milk. Camel milk may boost anti-proliferative effects and regulate antioxidant genes during cancer and hepatitis, hence lowering oxidative stress^11^. However, a meta-analysis of published data on the gross composition of milk of one-humped and Bactrian camels and East African one-humped camels produced mean values in g/100 mL for the following components: fat content (3.82 ± 1.08), total protein (3.35 ± 0.62), lactose (4.46 ± 1.03), total solids (12.47 ± 1.53), and ash (0.79 ± 0.09) and fat content (4.14 ± 0.80), total protein (3.33 ± 0.52), lactose (4.18 ± 0.72), total solids (12.69 ± 1.11), and ash (0.76 ± 0.09), respectively^12^. Meanwhile, El-Loly et al.^13^ found 11.30, 4.40, 8.12, 2.91, 3.18, and 0.90% for total solids, fat, solids not fat, protein, lactose, and Ash of camel milk, respectively. An examination of milk fat using gas liquid chromatography revealed molar percent values of 26.7 for palmitic, 25.5 for oleic, 11.4 for myristic, and 11.0 for palmitoleic^14^. The camel milk protein rich in glutamic acid, proline, aspartic, therionine, valine, and leucine and lower in lysine, cystine, and methionine^13^. Moreover, Mani and Deepak^15^ reported camel milk rich source of vitamins, minerals, lactoferrin, immunoglobulin, and insulin.

One of the most popular complementary and alternative medicine modalities utilized globally is herbal medicine, also known as phytotherapy in Germany and traditional Chinese’s medicine^16^. Silymarin, a flavonolignan derived from the seeds of "milk thistle" (*Silybum marianum* (L.) Gaertn.), has been widely used due to its potent hepatoprotective properties. The mixture mainly consists of three flavonolignans (silidianin, silychristin, and silybin,silybin is the most active and has shown antioxidant, anti-inflammatory, anti-fibrotic, anti-lipid peroxidative, immunological stimulant, and hepatic cell stabilizing effects^17^. Silymarin has been used therapeutically to treat cirrhosis, acute and chronic viral hepatitis, toxin- or drug-induced hepatitis, as well as alcoholic liver diseases. It has also been said to be effective in treating certain cancers such as hepatocellular carcinoma^18^. Therefore, the aim of the present work was to study the sub-chronic toxic effects of AFB1 on male albino rats and the possible ameliorating effect of camel milk or silymarin against AFB1 toxicity by assessment of some blood and serum biochemical parameters, and liver histopathology. Moreover, the study investigated the effect of AFB1, camel milk, and silymarin on the gene expression of tumour necrosis factor (*TNF-α*), antioxidant genes (NAD(P)H quinone oxidoreductase 1 (*NQO1*)), and base excision repair genes (*APE1* and *OGG1*) in the liver tissue.

## Materials and methods

### Ethical approval

All experimental procedures were carried out according to the NIH general guidelines for the care and use of laboratory animals and as recommended by the Ethics of Animal Use in Research Committee (IACUC), Faculty of Veterinary Medicine, Alexandria University, Egypt (Serial Number: 0304593).

### Treatments

Aflatoxin B1 was obtained from the Mycology Department, Animal Health Research Institute, Giza, Egypt. In brief, *Aspergillus flavus* microbes (a toxic strain with gene bank accession number: KP137700) were isolated from commercial diets and used to produce aflatoxin B1 (AFB1). The production of AFB1 was performed at the Mycology Department, Animal Health Research Institute, Giza, Egypt. The malt agar was used for the subculture and growth of the toxigenic strain for a week. Then toxigenic *A. flavus* strains were cultured on yeast sucrose broth for 21 days at 28–30 °C, according to Shotwell et al.^19^, Thin Layer Chromatography (TLC). The emergence of blue fluorescence on the plate and a comparison of the spot's Retention Factor (RF) value to that of a known standard were used to validate the presence of different AFs qualitatively. The gross and TLC examinations verified that commercially crushed yellow corn was completely free of fungus or mycotoxins contaminations. The commercially crushed yellow corn was autoclaved at 121 °C for fifteen minutes in the conical flasks for 3 successive days. After that, the autoclaved corn was treated with 10 ml of the spore suspension of the toxigenic strain (106 spores/ml). Then the treated corn was fermented by incubation at 28–30 °C for twenty-one days. After 21 days, the fungus in the incubated corn was killed in a 50 °C oven for 3–4 days. After that, the grinder was used to powder the crushed corn. Then 25 g of powdered corn as a representative sample was used to calculate AFB1^20^. Then the contaminated corn was incorporated into the commercially crushed corn to provide the desired dose of 1.4 mg of aflatoxin /kg of diet.

Camel milk was obtained from a local market in Marsa Matrouh, Egypt. The camel milk was transported to the laboratory in bottles and maintained there with ice. Meanwhile, silymarin was obtained from Hepamarin capsules (140 mg silymarin/capsule, batch no. for Silamarin is1122665) (UNIPHARMA is the abbreviation name of “Universal Industries Pharmaceuticals Company” located in the industrial area of Al-Obour city near Cairo, Egypt.).

### Experimental animals and design

**Figure 1:**
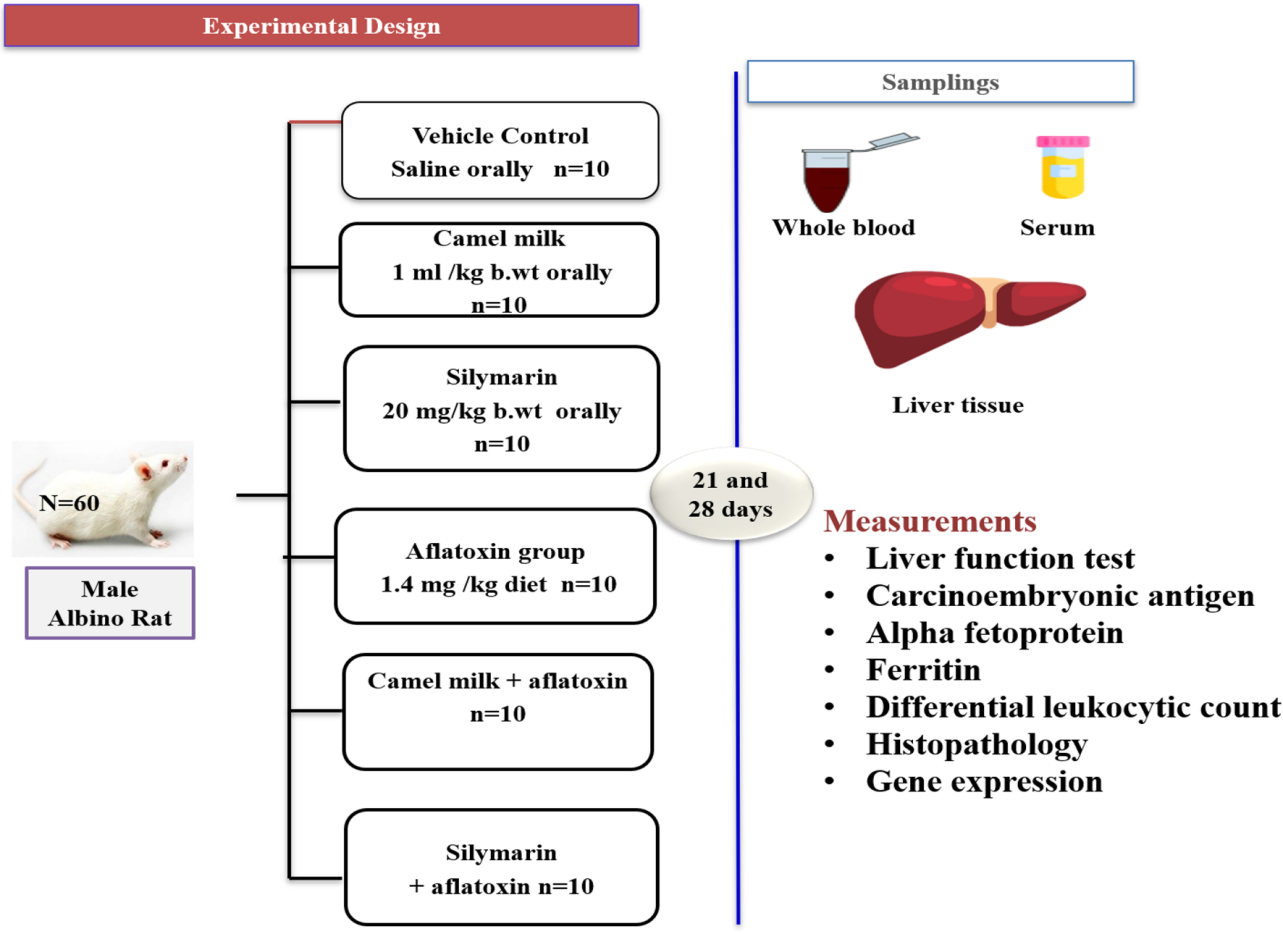
The experimental design.

A total of sixty mature male Wister white rats with an average weight of 100 ± 20 g for each rat were used to conduct this study. The animals were obtained from a private farm, Alexandria, Egypt. The experimental animals were kept in ploy propylene cages (70 × 50 × 30 cm) with an adjusted temperature of 19–22 °C, relative humidity 60%, and 12 h light–dark cycle. Throughout the experiment, all rodents were given free access to filtered water and normal rodent food. The rats were acclimatized to our laboratory conditions for two weeks before the experimental procedure. Figure 1 shows the six experimental groups (ten rats/group). The control group (without any treatment) received saline by gavage. The camel milk group received 1 ml of camel milk/kg body weight^21^ by gavage. The silymarin group received 1 ml of silymarin suspension solution at a dose of 20 mg silymarin/kg b.wt.^22^ by gavage. The aflatoxin group received an aflatoxin-contaminated diet at a dose of 1.4 mg of aflatoxin/kg of diet^23^. The camel milk + aflatoxin group received the same previous oral doses of camel milk and an aflatoxin-contaminated diet at the same time. The silymarin + aflatoxin group received the same previous doses of silymarin orally and an aflatoxin-contaminated diet at the same time. Saline, camel milk, or silymarin, were given for 28 days at 10 AM.

### Sampling

Samples were collected from all groups after 21 and 28 days of treatment. The rats were anaesthetized with isoflurane. In two sterile vacutainer tubes, blood was drawn from the medial canthus of the eye. For blood collection, one contains EDTA (an anticoagulant), whereas the other does not. The collected samples were kept at − 20 °C until further use for chemical examination. After sample collection, rats were sacrificed by cervical dislocation. Liver tissue was washed in saline and divided into two parts: one put in formalin for histopathological examination and the other stored at − 80 °C until further use in RNA isolation.

### Hemato-biochemical analysis

The hematological profile involved total counts (TC) of WBC (white blood corpuscles) and differential counts (DC) of the WBC. The hematological parameters were estimated using the techniques described by Ritchie et al.^24^. Serum alanine aminotransferase (ALT), total protein, and albumin, as well as aspartate aminotransferase (AST), were measured calorimetrically by using commercial kits from Biosystems® and BIOLABO®, Egypt, respectively. Meanwhile, the quantitative determination of carcinoembryonic antigen (CEA), alpha-fetoprotein (AFP), and ferritin concentrations was done by a microplate immuno-enzymometric assay, according to Sikorska et al.^25^, Tietz^26^, and Burtis et al.^27^, respectively.

### Histological processing of the liver

The liver tissue was fixed in 10% neutral buffered formalin. The fixed organ was dehydrated in ascending concentrations of alcohol from 70% until reaching absolute alcohol, cleared using multiple changes of xylene, impregnated with paraffin wax, and embedded in paraffin wax using a mould of suitable size. Paraffin blocks containing specimens were then cut into thin sections (5 μM each) using a microtome, and selected sections were mounted on glass slides. The preparation of paraffin sections for staining included the removal of paraffin wax using xylene, the removal of xylene using absolute alcohol, treatment with a descending concentration of alcohol (from absolute to 70%), and washing the sections with distilled water. Sections were stained with "Hematoxylin and Eosin" (H&E) stain and examined under the light microscope^28^.

### Gene expression

Quantitative real-time PCR (qRT-PCR) was used to determine the expression of base excision repair genes (apurinic/apyrimidinic endodeoxyribonuclease 1 (*APE1*) and 8-oxoguanine DNA glycosylase (*OGG1*)), tumor necrosis factor α (*TNFα*), and a multi-functional antioxidant gene involved in xenobiotic detoxification (NAD(P)H quinone oxidoreductase 1 (*NQO1*)). The liver samples (n = 5) were collected, frozen in liquid nitrogen, and stored at -80 °C for further RNA extraction. Following the manufacturer's instructions, RNA was extracted from the frozen samples using an RNA Purification Kit from Thermo Scientific (Cat. no. K0732, USA). The Intron-Power cDNA synthesis kit (Thermo Scientific, Cat. no. 25011, USA) was used to create cDNA from a fixed concentration of RNA. For the qRT-PCR assay: specific primers were used to amplify *NQO 1*, *TNFα*, *APE1*, and *OGG1* in rats, with the β-actin housekeeping gene for normalization. The primers are presented in Table 1. The obtained data were analyzed using the 2^−ΔΔCt^ method^29^.

**Table 1:** The primers used in this study.

Genes	Primer’s sequence (5′–3′)	Acc. Number
*NQO 1*	F: ACCTCTCTGTGGTTTAGGGC	NM_017000.3^30^
	R: GGACCTGGGTGTGCTATGTA	
*TNFα*	F: CCACGTCGTAGCAAACCACCAAG	NM_012675.3^31^
	R: CAGGTACATGGGCTCATACC	
*APE1*	F: GCTCAGAGAACAAACTCCCG	XM_017599805.2^32^
	R: TTGTTTCCTTTGGGGTTACG	
*OGG1*	F: CCTGGCTGGTCCAGAAGTAG	XM_039108420.1^32^
	R: TTTCCCAGTTCTTTGTTGGC	
*β-actin*	F: CACCATGTACCCAGGCATTG	NM_031144.3^30^
	R: ACAGTCCGCCTAGAAGCATT	

*NQO 1* NAD(P)H quinone oxidoreductase 1, *TNFα* tumor necrosis factor α, *APE1* apurinic/apyrimidinic Endodeoxyribonuclease 1, *OGG1* 8-oxoguanine DNA glycosylase.

### Statistical analysis

The data were analyzed by one-way ANOVA followed by Duncan’s post hoc test for multiple group comparisons using GraphPad Prism version 7.00 for Windows, GraphPad Software, La Jolla, California, USA, www.graphpad.com. The results were mean ± SE significant statistically (*P* ≤ 0.05).

### Guidelines

All methods were carried out in accordance with relevant guidelines and regulations. The authors confirm that the study was carried out in compliance with the ARRIVE guidelines.

## Results

Table 2 shows the ameliorative effects of camel milk and silymarin on leukocytic count for rats fed on AFB1-contaminated diets (1.4 mg/kg diet) for 21 and 28 days. At 21 days, when compared to the control group, the WBC and neutrophil counts in the aflatoxicosis group were significantly lower. On the other hand, when compared to the control group, the eosinophil concentration in the aflatoxicosis group significantly increased. Meanwhile, lymphocyte and basophil concentrations decreased without being significant. Furthermore, when compared to the control group, the aflatoxicosis group’s MON count increased insignificantly. Regarding the results of the camel milk and silymarin groups, there was a significant increase in the WBC count and eosinophil and basophil concentrations in the camel milk group when compared with the control group. However, the treatments used (camel milk and silymarin) ameliorated the negative effects of aflatoxin on the leukocytic count.

**Table 2:** Effect of camel milk and silymarin on leukocytic count for rats fed on diet containing 1.4 mg aflatoxin B1/kg diet for 21 and 28 days.

Groups items	Day	Control	Camel milk	Silymarin	Aflatoxin	Aflatoxin + camel milk	Aflatoxin + Silymarin
WBCs count (× 10^3^/µl)	21	9.96 ± 0.34^bcX^	12.96 ± 0.55^aX^	10.08 ± 0.26^bX^	7.77 ± 0.45^dX^	10.38 ± 0.16^bX^	8.89 ± 0.32^cX^
	28	11.1 ± 0.28^cY^	15.24 ± 0.50^aY^	13.50 ± 0.05^bY^	10.10 ± 0.19^dY^	11.36 ± 0.41^cY^	11.04 ± 0.25^cY^
LYM (× 10^3^/µl)	21	74.60 ± 1.75^abX^	78.20 ± 1.32^aX^	70.40 ± 1.94^bX^	69.60 ± 1.21^bX^	76.80 ± 2.82^aX^	69.80 ± 1.02^bX^
	28	86.40 ± 1.33^aY^	87.00 ± 1.30^aY^	85.00 ± 2.43^aY^	69.60 ± 2.04^bX^	85.40 ± 1.50^aY^	83.40 ± 2.25^aY^
NET (× 10^3^/µl)	21	20.20 ± 1.56^bX^	22.60 ± 0.93^abX^	24.00 ± 1.00^aX^	16.80 ± 1.36^cX^	15.40 ± 0.40^cX^	22.20 ± 0.66^abX^
	28	9.00 ± 0.45^cY^	9.00 ± 0.32^cY^	9.4 ± 0.60^bcY^	23.40 ± 0.51^aY^	10.00 ± 0.45^bcY^	10.80 ± 0.58^bY^
ESI (× 10^3^/µl) 2	21	1.30 ± 0.03^cX^	1.90 ± 0.16^aX^	1.52 ± 0.06^bcX^	1.82 ± 0.11^abX^	2.02 ± 0.13^aX^	2.06 ± 0.10^aX^
	28	0.96 ± 0.10^cY^	1.88 ± 0.07^aX^	1.32 ± 0.09^bX^	1.16 ± 0.07^bcY^	1.16 ± 0.07^bcY^	1.30 ± 0.08^bY^
MON (× 10^3^/µl) 1	21	3.60 ± 0.24^bX^	4.60 ± 0.24^abX^	4.40 ± 0.51^abX^	4.00 ± 0.45^abX^	4.00 ± 0.32^abX^	5.00 ± 0.32^aX^
	28	3.40 ± 0.24^cX^	4.60 ± 0.40^aX^	3.80 ± 0.20^bcX^	2.60 ± 0.24^dY^	3.20 ± 0.20^cdX^	4.20 ± 0.20^abX^
BAS (× 10^3^/µl) 3	21	0.38 ± 0.04b^dX^	0.58 ± 0.04^cX^	0.48 ± 0.04^cdX^	0.30 ± 0.03^bX^	0.50 ± 0.04 ^cX^	0.74 ± 0.05^aX^
	28	0.24 ± 0.02^bX^	0.52 ± 0.02^aX^	0.48 ± 0.04^aX^	0.24 ± 0.02^bX^	0.24 ± 0.02^bY^	0.30 ± 0.04^bY^

Values are means ± standard error. Mean values with different subscript letters (a-d) at the same row significantly differ at (*P* ≤ 0.05). Mean values with different subscript letters (X–Y) at the same column significantly differ at (*P* ≤ 0.05).

*WBCs* white blood cells, *LYM* lymphocyte, *NET* neutrophil, *ESI* eosinophil, *MON* Monocytes, *BAS* basophil.

At 28 days, there was a significant decrease in WBC count, lymphocyte count, and monocyte count in the aflatoxicosis group when compared with the control group. On the other side, there was a significant increase in the neutrophil concentration in the aflatoxicosis group when compared with the control group. Regarding the results of the camel milk and silymarin groups, there were significant increases in the WBC count, eosinophil count, and basophil count in both treatment groups when compared with the control group. Meanwhile, monocyte numbers were significantly higher in the camel milk group when compared with the control group. However, the treatments used (camel milk and silymarin) ameliorated the negative effects of aflatoxin on the leukocytic count.

Regarding results from different periods inside the same group, the results of the WBCs and lymphocytes were significantly increased in the control group, camel milk, and silymarin groups at 28 days when compared with results at 21 days. Meanwhile, neutrophils and eosinophils were significantly decreased at 28 days compared with 21 days, but monocytes and basophils were not affected by the period in the control group, camel milk, or silymarin groups. However, eosinophils were decreased in the camel milk and silymarin groups, but not significantly. On the other hand, the results of the aflatoxicosis group showed a significant decrease in the WBC count, neutrophils, eosinophils, and monocytes at 28 days when compared with 21 days. Meanwhile, lymphocytes and basophils were not affected by the period. The treatments used, on the other hand, improved the leukocytic count with the periods.

Table 3 shows the effects of camel milk and silymarin on serum biochemistry for rats fed on AFB1-contaminated diets (1.4 mg/kg diet) 21 and 28 days. At 21 days, there was a significant increase in the activities of ALT and AST and the concentrations of alpha fetoprotein and carcinoembryonic antigen in the aflatoxicosis group when compared with the control group. On the other side, there was a significant decrease in the total protein and ferritin concentrations without any effect on the other serum proteins in the aflatoxicosis group when compared with the control group. Regarding the results of the camel milk and silymarin groups, there was a significant increase in ferritin and ferritin and total protein concentrations in the camel milk and silymarin groups, respectively, when compared with the control group. However, the treatments used (camel milk and silymarin) ameliorated the negative effects of aflatoxin on serum biochemical parameters.

**Table 3:** Effect of camel milk and silymarin on some biochemical values for rats fed on diet containing 1.4 mg aflatoxin B1/kg diet for 21 and 28 days.

Groups items	Day	Control	Camel milk	Silymarin	Aflatoxin	Aflatoxin + camel milk	Aflatoxin + Silymarin
ALT (U/L)	21	57.60 ± 1.57^cX^	53.80 ± 2.35^cX^	56.20 ± 0.80^cX^	71.40 ± 1.86^aX^	55.00 ± 1.52^cX^	66.40 ± 1.44^bX^
	28	76.0 ± 1.41^bY^	56.20 ± 0.97 ^dX^	65.4 ± 0.68^cY^	85.0 ± 1.26^aY^	59.2 ± 0.97^dX^	81.20 ± 2.43^Ay^
AST (U/L)	21	289.0 ± 1.22^cX^	263.8 ± 1.16^dX^	266.2 ± 2.60^dX^	330.0 ± 2.07^aX^	302.4 ± 2.04^bX^	290.0 ± 2.57^cX^
	28	306.4 ± 2.69^eY^	294.4 ± 2.16^fY^	329.4 ± 0.68^dY^	412.2 ± 2.65^aY^	373.2 ± 2.08^cY^	385.2 ± 1.93^bY^
T. Protein (g/dl)	21	7.79 ± 0.41^abX^	7.90 ± 0.18^abX^	8.25 ± 0.16^aX^	7.27 ± 0.12^bX^	7.30 ± 0.24^bX^	7.44 ± 0.09^bX^
	28	7.08 ± 0.07^abX^	7.55 ± 0.16^aX^	7.31 ± 0.21^abY^	6.93 ± 0.09^bX^	6.94 ± 0.30^bX^	7.30 ± 0.08^abX^
Albumin (g/dl)	21	4.13 ± 0.19^aX^	4.31 ± 0.34^aX^	4.51 ± 0.33^aX^	3.84 ± 0.1^aX^	4.04 ± 0.19^aX^	4.16 ± 0.14^aX^
	28	3.96 ± 0.13^abX^	4.39 ± 0.06^aX^	4.26 ± 0.06^aX^	3.63 ± 0.24^bX^	3.99 ± 0.12^abX^	3.99 ± 0.24^abX^
Globulin (g/dl)	21	3.14 ± 0.24^aX^	3.75 ± 0.37^aX^	3.74 ± 0.41^aX^	3.28 ± 0.15^aX^	3.59 ± 0.22^aX^	3.46 ± 0.22^aX^
	28	3.12 ± 0.17^aX^	3.31 ± 0.37^aX^	3.31 ± 0.19^aX^	2.67 ± 0.10^aX^	3.16 ± 0.21^aX^	3.30 ± 0.27^aX^
Ferritin (mg/dl)	21	1.50 ± 0.09^bX^	1.57 ± 0.13^bX^	1.63 ± 0.12^bX^	1.37 ± 0.07d^X^	1.42 ± 0.13^bX^	2.09 ± 0.07^aX^
	28	2.12 ± 0.26^bcY^	2.66 ± 0.22^abY^	2.97 ± 0.36^aY^	1.32 ± 0.10^bX^	1.55 ± 0.21^cdX^	1.55 ± 0.06^cdY^
Alpha fetoprotein (ng/mL)	21	5.57 ± 0.32^bX^	2.14 ± 0.44^cX^	5.39 ± 0.49^bX^	8.56 ± 0.12^aX^	4.74 ± 0.21^bX^	7.84 ± 0.15^aX^
	28	8.66 ± 0.16^bY^	1.46 ± 0.14^dY^	4.68 ± 0.26^cY^	10.56 ± 0.18^aY^	8.95 ± 0.37^bY^	10.13 ± 0.42^aY^
Carcinoembryonic antigen (CEA) ng/mL	21	0.07 ± 0.00^dX^	0.24 ± 0.00^cX^	0.09 ± 0.00 ^dX^	0.62 ± 0.02^aX^	0.29 ± 0.01^bX^	0.40 ± 0.02^dX^
	28	0.51 ± 0.02^By^	0.25 ± 0.02^cY^	0.26 ± 0.01^cY^	0.86 ± 0.06^aY^	0.53 ± 0.03^bY^	0.58 ± 0.02^bY^

Values are means ± standard error. Mean values with different subscript letters (a–f) at the same row significantly differ at (*P* ≤ 0.05). Mean values with different subscript letters (X–Y) at the same column significantly differ at (*P* ≤ 0.05).

At 28 days, there was a significant increase in AST, ALT, alpha fetoprotein, and carcinoembryonic antigen in the aflatoxicosis group when compared with the control group. On the other side, there was a significant decrease in the total protein, albumin, and ferritin concentrations in the aflatoxicosis group when compared with the control negative group. Regarding the results of the camel milk and silymarin groups, there were significant increases in ferritin and decreases in AST, ALT, alpha fetoprotein, and carcinoembryonic antigen in both treatment groups when compared with the control group. However, the treatments used (camel milk and silymarin) ameliorated the negative effects of aflatoxin on serum biochemical parameters.

Regarding results from different periods inside the same group, the results of the serum parameters were significantly increased in all groups at 28 days when compared with results at 21 days, except that serum protein was not affected, and ferritin was increased.

**Figure 2:**
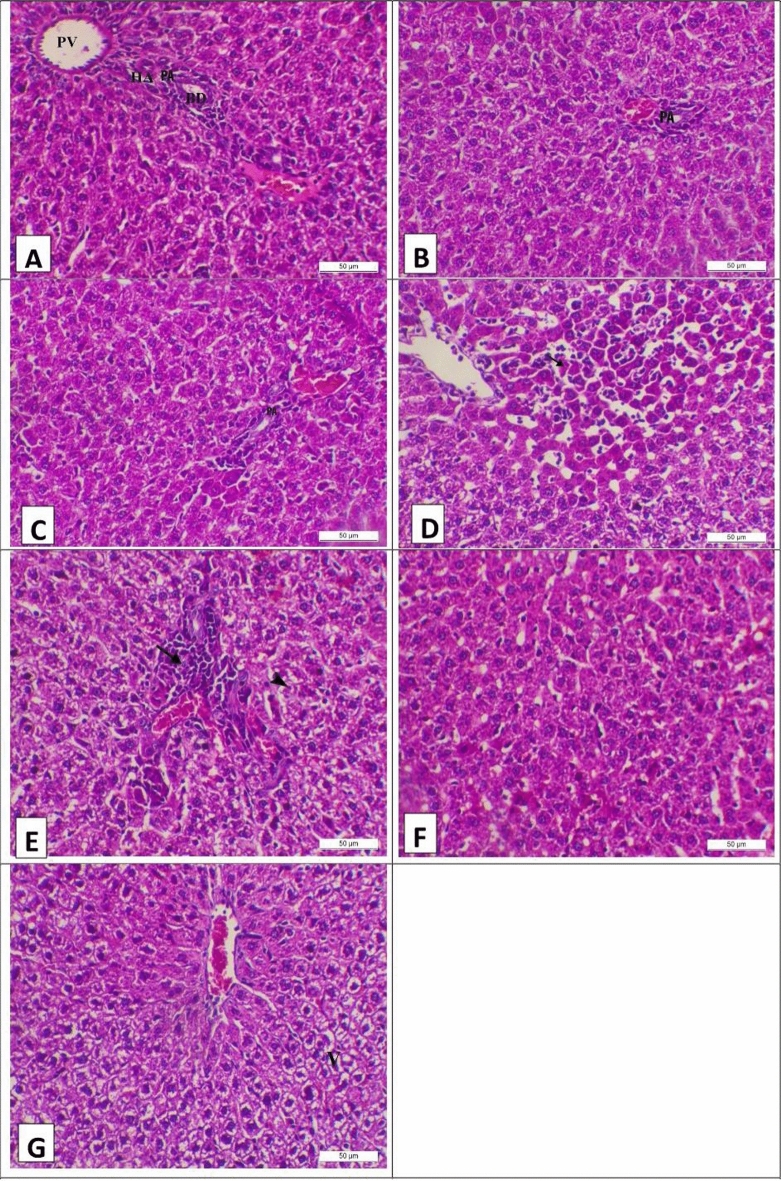
Photomicrograph of the liver of experimental groups at the 21th day of the experiment stained with H&E (X200, Scale bar = 50 µ) (**A**) control group showing normal hepatic histology featuring normal hepatocytes arranging in cords and normal portal area (PA) including portal vein (PV), hepatic artery (HA) and bile duct (BD). (**B**) Camel milk group showing normal hepatic histology featuring normal hepatocytes arranging in cords and normal portal area (PA). (**C**) Silymarin group showing normal hepatic histology featuring normal hepatocytes arranging in cords and normal portal area (PA). (**D**) Aflatoxin group showing coagulative necrosis of the centrilobular hepatocytes with mononuclear cells infiltration (arrow). (**E**) Aflatoxin group showing marked mononuclear cells infiltration (arrow) in the portal area with diffuse vacuolar degeneration of the midzonal hepatocytes (arrow head). (**F**) Aflatoxin and camel milk group showing normal hepatic histology. (**G**) Aflatoxin and Silymarin group showing vacuolar degeneration of the midzonal hepatocytes (V).

**Figure 3:**
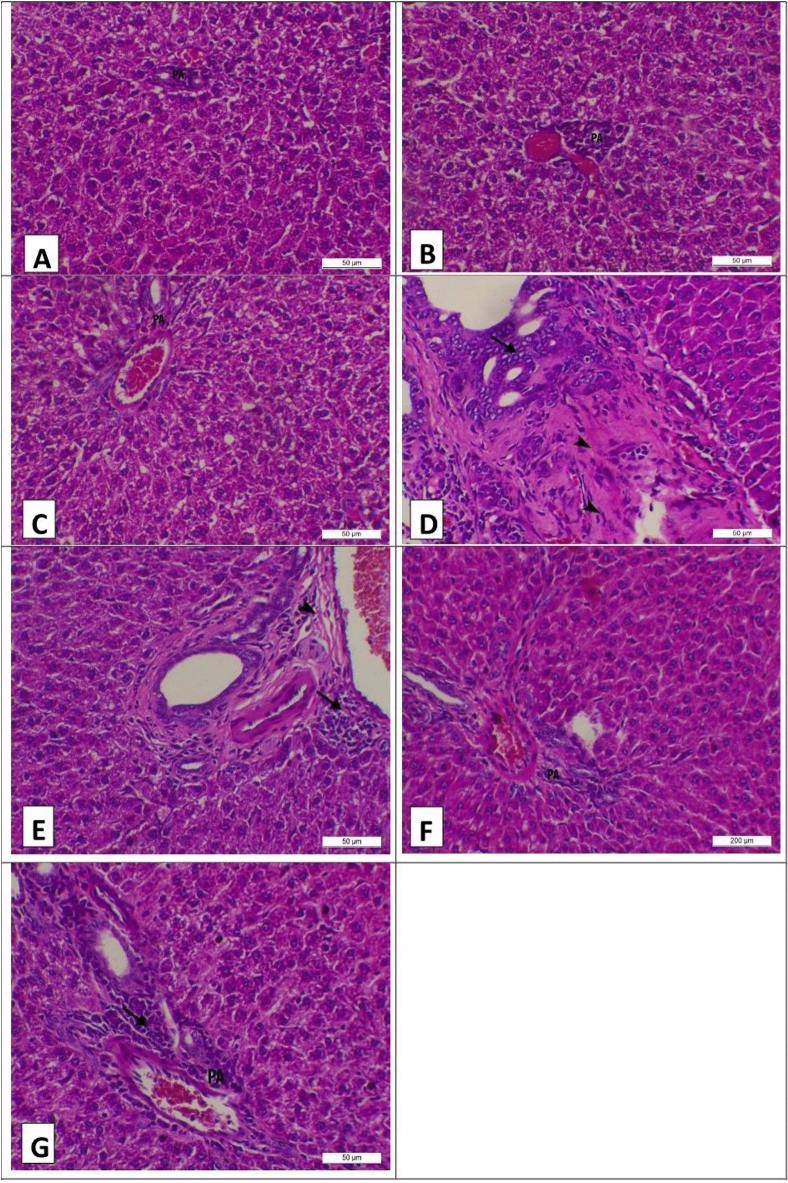
Photomicrograph of the liver of experimental groups at the 28th day of the experiment stained with H&E (X200, Scale bar = 50 µ) (**A**) Control group showing normal hepatic histology featuring normal hepatocytes arranging in cords and normal portal area (PA). (**B**) Camel milk group showing normal hepatic histology featuring normal hepatocytes arranging in cords and normal portal area (PA). (**C**) Silymarin group showing normal hepatic histology featuring normal hepatocytes arranging in cords and normal portal area (PA). (**D**) Aflatoxin group showing marked fibrosis of the portal area (arrow heads) and many newly formed bile ducts (arrow). (**E**) Aflatoxin group showing marked fibrosis of the portal area (arrow head) and intense mononuclear cells infiltration in the portal area (arrow). (**F**) Aflatoxin and camel mild group showing normal hepatocytes and portal area (PA). (**G**) Aflatoxin and silymarin group showing normal hepatocytes and mild mononuclear cells infiltration (arrow) in the portal area (PA).

Regarding the results of the liver histopathology at different periods (21 and 28 days), they are shown in Figs. 2 and 3. The liver of the control group at 21 days from the experimental period showed normal hepatic histology, featuring normal hepatocytes arranged in cords and a normal portal area (PA), including the portal vein (PV), hepatic artery (HA), and bile duct (BD) (Fig. 2A). On the other hand, livers of the camel milk and silymarin groups at 21 days from the experimental period showed normal hepatic histology featuring normal hepatocytes arranged in cords and a normal portal area (PA) (Fig. 2B and C, respectively). Meanwhile, the liver of the aflatoxin group at 21 days from the experimental period showed coagulative necrosis of the centrilobular hepatocytes with mononuclear cell infiltration (arrow) (Fig. 2D). Moreover, Fig. 2E shows marked mononuclear cell infiltration (arrow) in the portal area with diffuse vacuolar degeneration of the midzonal hepatocytes (arrowhead) in the same previous group. However, camel milk mitigates the bad effects of aflatoxin, with the livers of the aflatoxin and camel milk group showing normal hepatic histology (Fig. 2F). Meanwhile, the aflatoxin and silymarin group showed vacuolar degeneration of the midzonal hepatocytes (V).

On the other hand, Fig. 3 showed the photomicrograph of the liver of the experimental groups on the 28th day of the experiment, with the control, camel milk, and silymarin groups showing normal hepatic histology featuring normal hepatocytes arranged in cords and a normal portal area (PA) (Fig. 3A, B, and C, respectively). Meanwhile, the aflatoxin group showed marked fibrosis of the portal area (arrow heads), many newly formed bile ducts (arrows), and intense mononuclear cell infiltration in the portal area (arrows) (Fig. 3D and E). However, camel milk that was used in aflatoxin showed normal hepatocytes and the portal area (PA) (Fig. 3F). But aflatoxin and silymarin showed normal hepatocytes and mild mononuclear cell infiltration (arrow) in the portal area (PA) (Fig. 3G).

**Figure 4:**
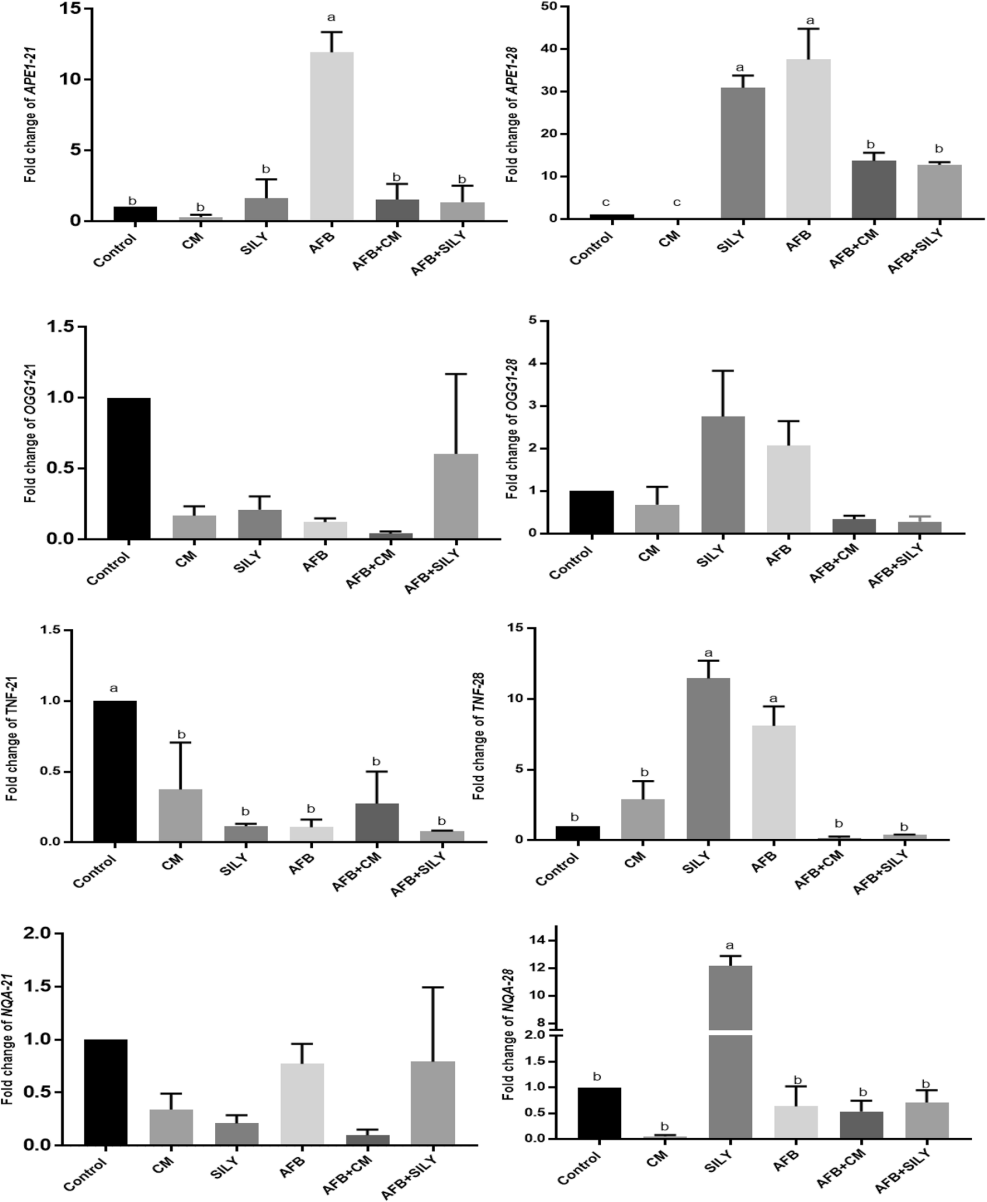
Ameliorative effects of camel milk and silymarin on (**A**) *APE1*, (**B**) *OGG1*, (**C**) *TNFα*, and (**D**) *NQO 1* gene expression in liver tissues.

Regarding the results of gene expression, qRT-PCR was used to evaluate gene expression levels, with normalization to *β-actin* mRNA at different periods (21 and 28 days), as shown in Fig. 4. At 21 days, aflatoxin administration produced a significant upregulation of *APE1* mRNA expression, a significant downregulation of *TNFα* and *OGG1*, and an upregulation of *NQO 1* without being significant in comparison with the control group. Meanwhile, the administration of camel milk and silymarin with aflatoxin significantly downregulated the gene expression of APE1 in comparison with the levels in the aflatoxin treated group, there was no significant downregulation of *OGG1* and *NQO 1* and significant downregulation of *TNFα* in comparison with the levels in the control group. At 28 days, silymarin alone and aflatoxin administration produced a significant upregulation of *APE1* and *TNFα* mRNA expression and an upregulation of *OGG1* without significant differences in comparison with the levels in control untreated rats. Meanwhile, the administration of camel milk and silymarin with aflatoxin significantly downregulated the gene expression of *APE1*, *TNFα*, and *NQO 1* with no significant downregulation of *OGG1* in comparison with the levels in the aflatoxin treated group.

## Discussion

The adverse changes in hepatic and renal biochemical parameters are often suggestive of the aflatoxin associated hepatic injuries in animals^33^. Even minute quantities of AF are harmful to animal health due to their negative impact on certain biochemical and haematological values^34,35^. The toxic effect of AF on hematology was clearly observed in this study and are in agreement with the findings of Kılıç et al.^36^ and Uluişik et al.^37^ who stated the same result when aflatoxin was given orally. Moreover, Abd Allah et al.^38^ and Mogilnaya et al.^39^ reported that AF induced a significant decrease in total and differential leukocytic counts, and lymphocytes. These outcomes could be explained by the hematopoietic tissue damage caused by aflatoxin^40^. On the other hand, Ahmed et al.^41^ reported that AFB1 increasde in the leukocytic count of rats. According to research, varying doses of AFB1 can either activate or suppress an organism's immune system^42,43^. However, these findings are assessed, as an illustration, of the immunosuppressive impact of aflatoxin B1^44,45^.

The negative effects of AFB1 on hematology were mitigated by camel milk and silymarin, which were orally ingested. An excellent protein source in camel milk has substantial biological effects that are associated with a decrease in infectious diseases^12^. Khan^46^ reported that consumption of camel milk mitigated the leukopenic effect of cyclophosphamide in mice. Moreover, Khazaei et al.^47^ reported that the WBC count rose when silymarin was included in the quail diet. El Elaimy et al.^48^ reported silymarin's therapeutic and preventative effects on rat immune toxicity brought on by chlorpyrifos. Camel milk is full of vitamins, minerals, and antioxidants that have become quite popular in the fight against xenobiotic toxicants^49,50^.

The primary location for xenobiotic metabolism is the liver^51^ and it has been assessed through recent studies that the AFB1 can be easily detected from the hepatic tissues of the infected animals^52^. It is regarded as one of the crucial body organs because of how it detoxifies or gets rid of pollutants and foreign materials^53^. These effects of AFB1 on the liver were reflected in the results of the serum biochemistry, especially the results of ALT, AST, and serum proteins. Serum enzyme activity results concur with those of El-Bahr^54^, Abdel-Wahhab et al.^55^, and Hatipoglu and Keskin^56^, who showed that liver enzymes increased considerably in AFB1-intoxicated rats compared to control. Serum ALT and AST activity increases are recognized as diagnostic signs of liver damage. Increased levels of AST and ALT in the blood due to the loss of structural integrity of hepatocytes in the AF group signal that some liver damage has occurred. Elevated liver enzymes also suggest damaging to hepatic parenchymal cells. Because AST and ALT are only released into the bloodstream when the structural integrity of the liver is compromised, where they ordinarily reside in the cytoplasm of hepatocytes^56–58^. Regarding the results of serum proteins, according to El‐Bahr^54^, rats intoxicated with AFB1 had considerably lower total protein levels than the control group. These may be caused by AFB1, which interferes with the protein biosynthesis by forming adducts with DNA, RNA, and proteins, inhibiting RNA synthesis and DNA-dependent RNA polymerase activity, and causing endoplasmic reticulum degranulation^59^. It has been demonstrated in the past that aflatoxins reduce the amount of total protein in rabbits^60^, broilers^57^, and fish^61,62^. Moreover, the results of the serum ferritin indicated a significant decrease in the aflatoxin group. The outcomes of Stoltzfus et al.^63^ support these findings, who reported that serum iron decreased because of decreased serum ferritin. AFB1 caused total iron binding capacity, serum iron, utilized iron-binding capacity, and transferrin amounts to drop^64^. Moreover, Techapiesancharoenkij et al.^65^ found that rats fed AFB1 had lower levels of transferrin, and this was linked to lower levels of transferrin mRNA. The ability of AFB1 to cause a reduction in the concentration of transferrin and β-globulin, which are responsible for iron binding capacity, is likely what causes the decrease in UIBC linked with AFB1 exposure^66^. Thus, AFB1 causes hypochromic microcytic anemia, a prevalent form of iron deficiency anemia^64^. On the other hand, Abdel-Wahhab et al.^67^ reported that consuming mycotoxins caused a substantial drop in serum levels of total iron binding capacity and iron, but had no discernible effect on serum ferritin levels. However, this adverse effect was improved by camel milk and silymarin consumption. On the other hand, the increase in AFP and CEA levels is consistent with a prior study that found that consuming AFB1 could raise these levels in rats^68–71^. Specific indicators for liver cancer include CEA and AFP. AFB1 increases the production of reactive oxygen species, which damage DNA and result in carcinogenesis^72^. However, the adverse effects of AFB1 on serum biochemistry were mitigated with camel milk and silymarin consumption. These findings are supported by Wang et al.^73^ findings, which showed that camel milk protected mice's livers by lowering levels of ALT and AST. Camel milk exhibits antigenotoxic and anticytotoxic properties in mice treated with cisplatin^74^. These results may be attributed to the anti-inflammatory and antioxidant effects of camel milk. There is compelling evidence to support the notion that the pathogenesis of aflatoxicosis is thought to be primarily mediated by lipid peroxidation, excessive oxidant generation, insufficient antioxidant capacity in cells, and mitochondrial alteration^75–77^. There have been claims that camel milk has anti-allergic properties where allergies can be brought on by a variety of foods, particularly ruminant milk and milk products, and some severe food allergies can cause anaphylactic responses^78^. Moreover, Khazaei et al.^47^ and Abou-Shehema et al.^79^ reported the protective effect of silymarin on the livers of aflatoxicated quail and broilers, respectively, which appeared in a decrease in serum ALT and AST concentrations. Muhammad et al.^80^ achieved the same outcomes, reporting that silymarin supplementation increased the serum protein of broilers. On the other hand, according to Sakamoto et al.^81^, silymarin was unable to counteract the harmful effects of pollutants on the metabolism and functionality of laying quail.

The outcomes of the liver's histological study supported the serum biochemistry results. The results of the liver pathology at different periods are in agreement with the results of El‐Bahr^54^, who reported that rats given AFB1 displayed altered lobular architecture and mild to severe degenerative alterations in their livers, which were characterized by swelling and hepatocytes that seemed to be in vacuoles. A considerable number of scattered solitary necrotic cells (apoptotic cells) could be seen in most hepatocytes. In the group that consumed AFB1, the histological examination revealed damaged livers with intense portal and lobular inflammation accompanied by fibrosis, like those previously observed^82,83^. Hepatocytes of aflatoxicated rats underwent histopathological and ultrastructural investigations, and the results showed severe vacuolar degeneration and necrosis signs^84^. However, this effect was mitigated by camel milk and silymarin ingestion. In rats receiving carbon tetrachloride treatment, camel milk has a hepatoprotective impact against liver damage. As a result, camel milk may be used to safeguard the liver from the toxic impacts of carbon tetrachloride and other chemical agents^85^. Moreover, El Miniawy et al.^86^ reported that camel milk mitigated the side effects of cisplatin on the liver. In rats with ethanol-induced liver damage, camel milk could lessen some hepatocytes' deterioration^87^. On the other hand, Tsai et al.^88^ reported that silymarin may be able to speed up the healing of rodents' carbon tetrachloride-induced liver fibrosis. Kheiripour et al.^89^ reported the hepatoprotective effect of silymarin in diabetic rats.

The pathophysiology of liver damage depends heavily on the inflammatory cytokines, which are produced when different stimuli, including viral infection or toxin exposure, are exposed to the body^90^, such as *TNF-α*^91^. At 28 days in the current study, aflatoxin treatment caused a substantial elevation of *TNF-* gene expression. However, in camel milk and silymarin with aflatoxin, TNF- gene expression was significantly downregulated. These findings concur with those of Jebali et al.^92^, who reported that the expression of TNF-, Bcl-2, and their target proteins were upregulated by AFB1 or AFM1 when Male Balb/c mice were exposed to AFB1 or AFM1 orally. Li et al.^93^ reported that when broilers were given AFB1 in the diet, the serum and spleen of broiler chickens may express more IL6, TNF-, and IFN-mRNA as a result of AFB1. Meanwhile, *TNF* gene expression was downregulated significantly at 21 days when compared to control groups. These findings are in line with those of Jiang et al.^94^, who found that the duodenum, jejunum, and ileum of broilers in the AFB1 group generally showed a drop in the expression of *IL-2*, *IL-4*, *IL-6*, *IL-10*, *IL17*, *IFN-*, and *TNF-mRNA*. Rats exposed to AFB1 had their expression of *IL-4*, *IFN-γ*, and *TNF-α* reduced, according to research by Qian et al.^45^. The type, the dose, the duration of exposure, the susceptibility of each tissue and animal species, as well as other experimental variables, may all have an impact on the effects of AFB1 on cytokines (TNF), which is why the results are not definitive^94^. However, the production of *TNF*-, *IL-6*, *iNOS*, and *NF-B/p65* was markedly increased by AF in liver tissue, which may help to cause inflammation. Notably, after AF exposure, upregulation of mRNA expression of these inflammation-related genes was previously noted in several investigations^95–98^. The initial and the most significant inflammatory mediator in the development of inflammation is *TNF*^99,100^. Proinflammatory cytokines like *IL-6* and *iNOS* are activated by *TNF-* and *NF-B/p65* pathways, along with adhesion molecules that promote leukocyte recruitment to the location of inflammation^7,34,101–103^. In the livers of mice consuming camel milk, *SAA1*, *TGF-α*, *TNF-α*, and *LCN2* mRNA expression was down-regulated, according to Wang et al.^73^. Exosomes from camel milk generated from colostrum were given to HepaRG, which also showed the lowest expression of genes linked to inflammation^104^.

*APE1* and *OGG1* are base excision repair genes; *APE1* showed significantly higher upregulation in the aflatoxin treated group, while *OGG1* was downregulated insignificantly when compared to the control group. These findings concur with those of Liu et al.^105^, who demonstrated that the expression of the *BER* genes 8-oxoguanine glycosylase-1 (*OGG1*) and X-ray repair cross complementing group 1 (XRCC1) was considerably downregulated by AFB1. The expression of the *BER* gene, apurinic/apyrimidinic endonuclease 1 (*APE1*), was increased when AFB1 and MC-LR were combined. *NQO1* is crucial in defending healthy cells from oxidative damage and tumorigenesis. The data showing that disruption of the NQO1 gene or genetic variation enhanced the risk of chemical-induced toxicity and cancer paradoxically showed the antioxidant role of *NQO1* despite the biological functions of this "cell protector"^106,107^. *NQO1* was upregulated insignificantly in the aflatoxin treated group at 21 days when compared to other groups. These findings concur with Lin et al.’s^108^ findings that malignant HCC exhibited up-regulation of *NQO1* and down-regulation of *NQO2*. Moreover, *NQO1* was downregulated significantly in the aflatoxin treated group at 28 days when compared to the silymarin group. These results are in agreement with Wang et al.^109^, who showed that keap1, *Nrf2*, and downstream target genes like *SOD*, *CAT*, *HO-1*, and *NQO1* all have dramatically reduced mRNA levels in the liver of aflatoxicated mice. Rajput et al.^110^ reported *Nrf2*, *HO-1*, *GCLC*, *NQO1*, and *SOD1* gene expression were reduced in the liver of aflatoxicated mice. On the other hand, Korashy et al.^111^ reported the ability of camel milk to activate both the extrinsic and intrinsic apoptotic pathways as well as the capacity of camel milk to greatly reduce the activation of the cancer-promoting gene cytochrome P4501A1 (*Cyp1a1*) and to stimulate the gene NAD(P)H quinone oxidoreductase 1 (*NQO1*)^112^.

## Conclusion

The inclusion of AFB1 in the rat diet had a substantial impact on the leukocytic and deferential counts, AST and ALT activity, serum proteins, ferritin, alpha-fetoprotein, and carcinoembryonic antigen findings. These results were confirmed by the results of liver histopathology and the gene expression of some antioxidant genes in the liver tissue. However, the addition of camel milk and silymarin reduced the effects of AFB1 on the hematology and serum biochemistry, liver pathology, as well as gene expression of the assessed genes.

## Data Availability

The datasets used and/or analysed during the current study available from the corresponding author on reasonable request.
